# Extent and Nature of Television Food and Nonalcoholic Beverage Marketing in 9 Asian Countries: Cross-Sectional Study Using a Harmonized Approach

**DOI:** 10.2196/63410

**Published:** 2024-12-04

**Authors:** Tilakavati Karupaiah, Shah Md Mahfuzur Rahman, Juan Zhang, Naveen Kumar, Batjargal Jamiyan, Raj Kumar Pokharel, Elaine Quintana Borazon, Tharanga Thoradeniya, Nguyen Thi Thi Tho, Sally Mackay, Bridget Kelly, Boyd Swinburn, Karuthan Chinna, Enkhmyagmar Dashzeveg, Gild Rick Ong, Sreelakshmi Sankara Narayanan, Mohd Jamil Sameeha, Mohammad Ahsan Uddin, Yuxiang Tang, Naresh Kumar Sharma, Rishav Pokharel, Anna Christine Rome, V Pujitha Wickramasinghe, Phan Thanh Huy

**Affiliations:** 1 School of Biosciences Faculty of Health and Medical Sciences Taylor's University Subang Jaya Malaysia; 2 Food Security and Nutrition Impact Lab Taylor’s University Subang Jaya Malaysia; 3 Department of Health Promotion and Health Education Faculty of Public Health Bangladesh University of Health Sciences Dhaka Bangladesh; 4 School of Population Medicine and Public Health Peking Union Medical College & Chinese Academy of Medical Sciences Beijing China; 5 Chitkara School of Health Sciences Chitkara University Punjab India; 6 Department for Nutrition Research National Center for Public Health Ministry of Health Ulaanbaatar Mongolia; 7 Public Health Nutrition Section South Asia Infant Feeding Research Network (SAIFRN)-Nepal (A Nepal Chapter of Global SAIFRN Network) Lalitpur Nepal; 8 International Graduate Program of Education and Human Development College of Social Sciences National Sun Yat-sen University Kaohsiung Taiwan; 9 Department of Biochemistry and Molecular Biology Faculty of Medicine University of Colombo Colombo Sri Lanka; 10 Noncommunicable Disease (NCD) Control Department National Institute of Hygiene and Epidemiology Hanoi Vietnam; 11 School of Population Health The University of Auckland Auckland New Zealand; 12 Faculty of the Arts, Social Sciences and Humanities Early Start, School of Health and Society University of Wollongong Wollongong Australia; 13 Faculty of Business and Management UCSI University Cheras Malaysia; 14 Nutritional Sciences Programme, Centre for Community Health Studies Faculty of Health Sciences Universiti Kebangsaan Malaysia Kuala Lumpur Malaysia; 15 Academic Wing Institute of Public Health Dhaka Bangladesh; 16 Amity Institute of Biotechnology Amity University Rajasthan Jaipur India; 17 Department of Food Science and Nutrition College of Home Economics University of the Philippines-Diliman Metro Manila Philippines; 18 Department of Paediatrics Faculty of Medicine University of Colombo Colombo Sri Lanka

**Keywords:** children, Asian food marketing, television, unhealthy food, WHO nutrient profile model, World Health Organization, pediatrics, commercial, Asia, unhealthy, nutrition, diet, market, advertisement, food, beverage, consumption

## Abstract

**Background:**

Rising childhood obesity rates in Asia are adding risk for the future adult burden of obesity and noncommunicable diseases. Weak policies across most Asian countries enable unrestricted marketing of obesogenic foods and beverages to children. Television is the common medium for food marketing to reach this audience.

**Objective:**

This study aimed to assess the extent and nature of television food and nonalcoholic beverage marketing in 9 Asian countries (Bangladesh, China, India, Malaysia, Mongolia, Nepal, the Philippines, Sri Lanka, and Vietnam) with capacity building support from the International Network for Food and Obesity/Non-Communicable Disease Research, Monitoring and Action Support, who enabled harmonization of data collection method and content analyses.

**Methods:**

Advertised foods were categorized as permitted or not permitted based on the nutrient profile models established by the World Health Organization regional offices for South-East Asia (SEARO) and the World Health Organization regional offices for Western Pacific (WPRO). Overall rates of food advertisements (advertisements per hour per channel) and persuasive strategy use were analyzed along with comparisons between children’s peak viewing time (PVT) and non-PVT.

**Results:**

Cross-country comparisons, irrespective of country income level, indicated that not permitted food advertising dominated children’s popular television channels, especially during PVT with rates as per WPRO or SEARO criteria ranging from 2.40/2.29 (Malaysia) to 9.70/9.41 advertisements per hour per channel (the Philippines). Persuasive strategy rates were also comparatively higher during PVT. Sugar-sweetened beverages, sugar-containing solid foods, and high salt- and fat-containing snacks and fast foods were frequently advertised. Evaluation of the application of WPRO and SEARO nutrient profile models identified inconsistencies due to regional taste and cuisine variations across Asia.

**Conclusions:**

This study clearly showed that unhealthy food marketing through popular children’s television channels is widely occurring in Asia and is a clear breach of child rights. Evidence outcomes will benefit advocacy toward stronger policy regulations to control unhealthy food marketing and strengthen strategies to promote a healthier food environment for Asia’s children.

## Introduction

Rising childhood obesity rates are a global health issue, with Asia not immune to this trend. Almost half of the world’s children younger than 5 years of age who are overweight or obese are living in Asia [1], a region that is also rapidly facing an upsurge in the prevalence of noncommunicable diseases (NCDs) such as diabetes, cardiovascular disease, and hypertension [2]. Even low- and middle-income countries (LMICs) in Asia, dealing with undernutrition as the traditional public health challenge, are not exempt from childhood overweight and obesity, particularly in urban settings [1]. Childhood obesity predicts NCD development in adulthood [3,4].

A systematic review of evidence examining interventions to prevent childhood obesity in the Asian region revealed that interventions tended to focus on children’s school settings and targeted behavioral modification through nutrition or health education and physical activity sessions [5]. Focusing on such nutrition and physical activity promotion programs fails to account for the effects of the wider food environment, where unhealthy foods and beverages high in saturated and trans fats, added sugars and sodium (termed as high in fat, sugar, and salt [HFSS]), and usually highly processed [6] are highly available, accessible, and heavily promoted [7,8]. This food environment is consistent with growing transnational and regional food business across Asia [9].

Television advertising of unhealthy foods is a big driver of children’s exposure to unhealthy food marketing [10-12]. Without question, children who are highly exposed to powerful marketing of HFSS foods are vulnerable to negative food behaviors that are conducive to overweight and obesity development [13-15]. Increased exposure of children to the marketing of unhealthy foods increases purchase requests [13,14] and develops tastes, preferences, and habits [16-19] for these foods. The habituation of unhealthy foods and beverages in children through advertising exposure is suggested by the World Health Organization (WHO) to be linked to childhood obesity [20].

Expenditure on television advertising rose from the year 2000 in the Asia-Pacific to reach US $55,692 million in 2024 [21]. Free-to-air, cable, or satellite television is a major media source in Asia [22] and comparatively more accessible for low-income households than digital media [23]. Therefore, the dominant influence of food marketing is through children’s popular television channels, as observed in Malaysia [24], Thailand [25], and India [26]. Children’s exposure to food advertising on television across Asia is largely unchecked because of weak or nonexistent regulations [27]. Slow policy progress is perpetuated by the lack of local research to document the extent of the issue and to hold industries accountable for protecting children from exploitation [8]. Further, current evidence on television food advertising is largely limited to high-income countries, and efforts are needed to support monitoring the nature of this advertising in low-resource settings [28]. Although some data are available for selected countries in Asia, including Malaysia [24,29] and Korea [30], only data from Korea have been able to inform favorable policy design. A major limitation of these Asian studies was the lack of uniform methodology, with researchers adopting varying approaches to categorize nutritional profiles of advertised foods. An Asia-Pacific comparison [31] involving China, Korea, Indonesia, and Malaysia benefited from the use of one standard methodology for data sourcing, recording, and coding but benchmarked nutritional comparisons to core and noncore food criteria rather than a standardized WHO nutrient profile model (NPM).

Acknowledging these gaps, a situational analysis was warranted to explore the extent and nature of unhealthy food and nonalcoholic beverage marketing through television across Asia. Further, the protection of children from exploitation as defined in the Conventions on the Rights of the Child [32] and specifically from unhealthy food marketing [33] constitutes child rights, which emphasizes the need to conduct this research in Asia. Keeping this in mind, we report on the outcomes of a collaboration between 9 Asian countries comprising 7 LMICs (Bangladesh, India, Mongolia, Nepal, the Philippines, Sri Lanka, and Vietnam) and 2 upper- and middle-income countries (China and Malaysia) [34]. This collaboration adopted the International Network for Food and Obesity/Non-Communicable Disease Research, Monitoring and Action Support (INFORMAS) food promotion module’s television protocol [35], which harmonized data collection procedures to enable country comparisons on key variables measuring children’s exposure to unhealthy food marketing in television. INFORMAS is a global network of public-interest organizations and researchers aiming to monitor, benchmark, and support public and private sectors to foster healthy food environments and combat obesity, NCDs, and their associated inequalities [36]. INFORMAS also served as a research stakeholder by supporting intensive capacity building in implementing a methodology for achieving the project’s milestones.

## Methods

### Study Design

The 9 Asian countries in this collaborative study agreed to adopt the INFORMAS protocol for food promotion [35]. Briefly, this protocol requires country-specific contextual information to be collected through the recording of children’s popular television channel broadcasting in order to identify food advertisements embedded between and during programs. This approach did not include other types of marketing such as product placement in shows and sponsorship of television shows. Food advertisements covered both solid foods and nonalcoholic beverages.

The INFORMAS methodology was adapted to enable a standard recording format to fit across the diverse food cultures, seasonal events, and children’s schooling and holiday periods of each country. Further, the advent of COVID-19 introduced a disruption to obtaining country clearance, funding disbursements, and data recording schedules. This meant that data recording could not be conducted simultaneously for all countries.

### Ethical Considerations

Depending on individual country regulations, some countries were exempted from obtaining ethics approval (India, Malaysia, Nepal, and the Philippines). Ethical clearance was not required for these countries for studies not involving human or animal subjects. Ethics approval was required for Bangladesh (IPH/AW/IRB/2020-21/03, institutional review board of the Institute of Public Health, Dhaka), China (202023, institutional review board of the Chinese Centers for Disease Control and Prevention), Mongolia (253, Ethics Committee under the Ministry of Health), Sri Lanka (EC-20-047, Ethics Review Committee, Faculty of Medicine, University of Colombo), and Vietnam (IRB-VN01057/IORG0008555, institutional review board of the National Institute of Hygiene and Epidemiology).

### Sample Selection

#### Defining Children’s Age Limit

Information on audience viewing patterns is usually sourced from media monitoring services and typically reported for children’s age limit set <12 years [37,38]. However, for this study’s purpose, age was defined according to country-specific regulations and considering the definition of the United Nations’s Conventions on the Rights of the Child [6,32]. Most country teams set children’s age as up to 18 years, except for India (up to 14 years), Sri Lanka (up to 15 years), the Philippines (up to 17 years), and Malaysia (up to 19 years).

#### Defining Popular Television Channels

The top 3 children’s popular television channels for each country were selected based on children’s viewership data [35]. These data were available from commercial media monitoring companies in China (Kuyun), India (Broadcast Audience Research Council), Malaysia (Nielsen), Mongolia (Maxima Consulting), the Philippines (Nielsen), Sri Lanka (Kantar), and Vietnam (Nielsen). Whereas in Bangladesh, popular channels were determined based on expert opinions (n=22), comprising television channel experts, advertising experts, food marketing officials, and caregivers. While in Nepal, expert opinions (n=6) were representative of the Ministry of Information Technology and Communication, national television children’s program specialist, cable television service provider, the National Codex committee, and caregiver. Additionally, cross-sectional surveys on television viewing habits were conducted with children and caregivers in Bangladesh and Nepal (n=400 and 51, respectively). In China, findings from a digital survey on parents (n=193) of children and adolescents, aged 3-18 years, were used to validate the commercial data.

#### Defining Culinary Ingredients

Groceries such as rice, cooking oil, and seasonings were also being advertised in some countries during the sampled day’s program broadcasting. These items were targeted to homemakers, as they were also television viewers during children’s programs in Asia. Collaborating teams agreed to exclude these items from recorded data, as the intention was to count only packaged food and beverage products cognizant of children’s exposure, direct purchasing request, and consumption. A list of these ingredients is provided in Table S1 in Multimedia Appendix 1, and the rates for these ingredients are compared in Table S2 in Multimedia Appendix 1.

#### Defining Peak Viewing Time

Television viewership data were also used to define peak viewing time (PVT) in each country. PVT represented the top five 1-hour time slots across the broadcast day, separated between weekdays and weekend days [39].

#### Training

Group training was conducted via videoconferencing by the project management team (PMT) formed by TK, GRO, and SSN with INFORMAS faculty support from SM and BK, to harmonize methodology across countries, prior to data collection.

#### Recording Protocol

An adapted recording protocol was adopted by all countries, whereby (1) the recording period was within a 3-month period, (2) convenience sampling was used to record for 4 weekdays and 4 weekend days, (3) the 8 recording days were within normal schooling weeks and excluding any public or school holidays and special events (eg, elections), and (4) concurrent recording was performed for 3 selected top channels with a recording duration up to 18 hours per day (6 to 12 AM).

#### Verification of Recording Procedures

Recording procedures were verified individually by the PMT for each country in one-to-one videoconferencing sessions, with support by faculty to troubleshoot issues. All country teams met the minimum data collection requirement. Some teams completed recording in 2020 (China, Malaysia, and Vietnam). Four teams rerecorded in 2021 (Bangladesh, Mongolia, the Philippines, and Nepal) due to limited viewership data access, school reopening after the COVID-19 lockdown, and data corruption issues. India and Sri Lanka teams completed recording after mid-2022 due to delays in obtaining their country clearance. The recording was performed by most countries using their own research teams, with the exception of outsourcing to providers by China (Kuyun) and Mongolia (Maxima Consulting) teams.

### Coding for Healthy Versus Unhealthy Foods

#### Overview

Each country team assigned at least 2 researchers (student, research assistant, or coinvestigator) to perform the coding of recorded food advertisements. Ongoing support was provided by the PMT and INFORMAS faculty. The primary coding steps involved (1) coding variables for broadcast television channel, date, day, program name or category, and time slot. The time slot variable was classified into PVT or non-PVT. (2) Categorizing all advertisements in the recorded data into food and nonfood items. Advertised food products required brand information (name, description, and parent company). (3) Coding recorded data of advertised foods and beverages according to the NPMs proposed by the World Health Organization regional offices for Western Pacific (WPRO) [40] and World Health Organization regional offices for South-East Asia (SEARO) [41]. The NPMs allow for the classification of foods that should be “permitted” and “not permitted” to be marketed to children based on nutrient thresholds [40,41]. Both models were used, as the participating countries spanned both regions. Additionally, the INFORMAS food classification system was adopted for food products not included in the NPM classifications, such as infant formula, alcohol, and dietary supplements [35]. (4) Coding up to the 3 most prominent food products, as defined by INFORMAS protocol [35], where multiple food products were being promoted in an advertisement. In cases where coders were unable to determine the level of prominence, coding priority was based on the first products shown or coding from the top-left quadrant of the food advertisements for the purpose of sequencing [35]. (5) Conducting market surveys, preferably in physical stores or through digital resources in each country, to retrieve an advertised product’s nutrient values to facilitate nutrient profiling. Products without nutrition information were labeled as “insufficient nutrition information panel.” Products that were not covered by the WHO NPMs (eg, 1- to 3-year follow-up milk formulae) were identified as “not applicable.” (6) Excluding data for banner advertisements and product placements during programs.

#### Reliability Testing

Intercoder reliability (IRR) testing within each country was performed by researchers who were responsible for the dataset coding. This required a randomly selected hour of television recording for testing reliability between coders according to the formula [35]:

Agreement/(agreement+disagreement)×100

The minimum required IRR score was 90% agreement between researchers. If this was not achieved, then additional training was provided to the coder before retesting coding reliability with another television recording sample. Once the minimum IRR was achieved within a country, then IRR testing was conducted between countries. This required each country to submit coded data to the PMT for a random hour of recording. The PMT’s coding for this dataset served as a comparator. The minimum required score was 80% agreement, and if this was not achieved, then a second or even a third IRR testing trial was conducted for a different hour of recording, following further training provided to the concerned country’s primary coder.

### Coding for Persuasive Power Strategies

Additional coding was performed for persuasive power strategies of marketing, comprising the use of power strategies and premium offers [35]. Up to 3 strategies per food advertisement were recorded.

### Data Processing

#### Cleaning

Upon completion of data entry, cleaned Microsoft Excel datasets were provided to the PMT for cross-checking. Country teams corrected datasets based on feedback. Differences were resolved through discussion with the PMT and INFORMAS faculty.

#### Aggregation

Data were weighted based on weekdays and weekend days. Advertisements were aggregated into hourly time slot intervals. Factors explored for advertisement rate analysis were (1) children’s viewing time (PVT vs non-PVT), (2) marketing permissibility (permitted vs not permitted), and (3) the use of persuasive power strategies.

### Statistical Analysis

Statistical analysis was performed using SPSS (version 26.0; IBM Corp). A data analysis training workshop (KC, SM, and BK) was conducted for country teams. The PMT checked and consolidated all countries’ datasets, before performing the final country comparator analyses.

Outputs for descriptive analysis to understand the extent of television food marketing were reported as mean (SD) for rate parameters (advertisements per hour per channel) applicable to all food advertisements, permitted food advertisements and not permitted food advertisements, and their ratios (permitted:not permitted) derived according to both WPRO and SEARO NPMs. Wilcoxon signed rank tests assessed comparisons between permitted versus not permitted food advertisements. Additionally, the Mann-Whitney *U* test assessed rate comparisons between PVT and non-PVT for not permitted food advertisements. Persuasive marketing strategies engaged for not permitted food advertisements, and interpreted as power strategies and premium offers, were also similarly assessed. Frequency analysis was then performed to understand the popularity of categories of not permitted food advertisements promoted on television according to WPRO and SEARO NPMs. This required coded items to be further consolidated to rank the top 5 most frequently advertised not permitted food categories (%). The significance threshold for all analyses was set at *P*<.05.

## Results

### Country Comparisons of Food Advertisement Rates for Permitted and Not Permitted Foods

For all 9 Asian countries investigated, food advertisement rates for not permitted foods were significantly higher (all *P*<.05) compared to permitted foods (Table 1). This trend was consistent, whether applying the criteria of the WPRO or SEARO NPMs. Advertisement rates for not permitted foods as per WPRO criteria were highest for the Philippines followed by Sri Lanka>Mongolia>India>Bangladesh>China>Nepal> Vietnam>Malaysia. This trend was also repeated when applying the SEARO criteria, except for not permitted food advertisement rates for China being greater than Bangladesh. However, comparisons between countries regarding the concentration of not permitted food advertisement rates, interpreted as ratio of permitted:not permitted advertisements, indicated that they were lowest for Sri Lanka and China, whereas the ratio of permitted:not permitted food advertisements was highest for Malaysia and Vietnam due to fewer permitted foods being promoted. Of interest, ratios for India and Bangladesh were higher with WPRO criteria than with SEARO criteria.

**Table 1:** Food advertisement rates for Asian countries as per World Health Organization regional offices for Western Pacific (WPRO) and World Health Organization regional offices for South-East Asia (SEARO) criteria.

Country	All food advertisements^a^	Food advertisement rates (advertisements per hour per channel)								
		WPRO					SEARO			
		Permitted, mean (SD)	Not permitted, mean (SD)	*P* value	Permitted:not permitted	Permitted, mean (SD)		Not permitted, mean (SD)	*P* value	Permitted:not permitted
Bangladesh	8.17 (8.32)	0.43 (1.21)	6.23 (6.67)	<.001	1:14.5	0.72 (1.29)		4.25 (6.00)	<.001	1:5.9
China	11.53 (10.07)	2.13 (2.84)	5.03 (8.57)	<.001	1:2.4	1.57 (2.07)		5.49 (9.60)	<.001	1:3.5
India	9.31 (5.97)	0.46 (0.89)	7.75 (5.36)	<.001	1:16.8	2.08 (2.39)		5.73 (4.56)	<.001	1:2.8
Malaysia	4.07 (6.48)	0.01 (0.11)	2.40 (3.88)	<.001	1:240	0.19 (0.50)		2.29 (3.71)	<.001	1:12.1
Mongolia	11.37 (10.80)	0.72 (1.30)	7.97 (7.67)	<.001	1:11.1	0.72 (1.83)		7.91 (7.46)	<.001	1:11.0
Nepal	4.62 (4.58)	0.11 (0.36)	3.89 (4.06)	<.001	1:35.4	0.11 (0.36)		3.47 (3.64)	<.001	1:31.5
Philippines	15.00 (15.12)	0.75 (1.18)	9.70 (9.85)	<.001	1:12.9	0.93 (1.38)		9.41 (9.47)	<.001	1:10.1
Sri Lanka	16.68 (11.26)	3.43 (3.55)	9.08 (6.84)	<.001	1:2.6	3.15 (3.29)		9.37 (7.16)	<.001	1:3.0
Vietnam	3.34 (6.86)	0.02 (0.15)	2.69 (5.77)	<.001	1:134.5	0 (0)		2.74 (5.90)	<.001	—^b^

^a^All food advertisements include solid foods and beverages advertised. Includes advertisements for culinary ingredients, nutritional supplements, baby food, and follow-up formula. Additionally, the term also covers advertisements for food companies, retailers, and outlets that do not promote specific food products.

^b^For Vietnam, permitted:not permitted could not be determined with SEARO criteria.

### Extent of Food Advertisements During PVT and Non-PVT

We further measured the appearance of not permitted food advertisements during PVT and non-PVT for all 9 countries (Table 2). For most countries, not permitted food advertisement rates occurring during PVT were significantly higher compared to rates during non-PVT regardless of both NPMs. For India and Nepal, there was no difference in the rates of not permitted food advertisements between PVT and non-PVT irrespective of both NPM criteria. For China, the rates of not permitted food advertisements when classified using the SEARO model were significantly higher during non-PVT. The difference observed for China as per WPRO (1.45 advertisements per hour per channel) and SEARO (1.29 advertisements per hour per channel) remained comparable but not significant.

**Table 2:** Appearance of not permitted food advertising during peak and nonpeak viewing times on television.

Country	Not permitted food advertisement rates^a^ (advertisements per hour per channel)					
	WPRO^b^			SEARO^c^		
	PVT^d^, mean (SD)	Non-PVT, mean (SD)	*P* value	PVT, mean (SD)	Non-PVT, mean (SD)	*P* value
Bangladesh	9.31 (8.00)	5.04 (5.65)	<.001	6.73 (7.11)	3.30 (5.22)	<.001
China	3.98 (5.80)	5.43 (9.40)	.24	4.56 (6.60)	5.85 (10.51)	.02
India	7.54 (4.34)	7.84 (5.70)	.86	5.70 (3.92)	5.75 (4.78)	.38
Malaysia	3.58 (4.76)	1.95 (3.37)	<.001	3.45 (4.72)	1.85 (3.14)	<.001
Mongolia	11.19 (8.60)	6.62 (6.81)	<.001	10.99 (8.33)	6.62 (6.66)	<.001
Nepal	3.67 (3.80)	3.97 (4.15)	.53	3.38 (3.51)	3.50 (3.69)	.84
Philippines	12.26 (10.87)	8.71 (9.24)	<.001	11.84 (10.47)	8.48 (8.89)	<.001
Sri Lanka	11.35 (6.31)	8.21 (6.84)	<.001	11.55 (6.21)	8.53 (7.32)	<.001
Vietnam	5.66 (7.18)	1.54 (4.65)	<.001	5.62 (7.32)	1.63 (4.81)	<.001

^a^Not permitted food advertisements include solid foods and beverages. It excludes advertisements for culinary ingredients, nutritional supplements, baby food, and follow-up formula.

^b^WPRO: World Health Organization regional offices for Western Pacific.

^c^SEARO: World Health Organization regional offices for South-East Asia.

^d^PVT: peak viewing time.

With both NPM criteria, not permitted food advertisement rates during PVT compared to non-PVT were nearly 1.7 times higher for Mongolia (WPRO: mean 11.19, SD 8.60 vs mean 6.62, SD 6.81 advertisements per hour per channel and SEARO: mean 10.99, SD 8.33 vs mean 6.62, 6.66 advertisements per hour per channel), nearly 2 times higher for Malaysia (WPRO: mean 3.58, SD 4.76 vs mean 1.95, SD 3.37 advertisements per hour per channel and SEARO: mean 3.45, SD 4.72 vs mean 1.85, SD 3.14 advertisements per hour per channel) and Bangladesh (WPRO: mean 9.31, SD 8.00 vs mean 5.04, SD 5.65 advertisements per hour per channel and SEARO: mean 6.73, SD 7.11 vs mean 3.30, SD 5.22 advertisements per hour per channel), and nearly 3 times higher for Vietnam (WPRO: mean 5.66, SD 7.18 vs mean 1.54, SD 4.65 advertisements per hour per channel and SEARO: mean 5.62, SD 7.32 vs mean 1.63, SD 4.81 advertisements per hour per channel).

### Defining Engagement of Persuasive Strategies in Not Permitted Food Advertisements

We conducted content analyses to probe the nature of engagement used by persuasive strategies for not permitted food advertisements targeting children in the context of power strategies and premium offers, with comparisons during PVT and non-PVT (Table 3).

Rates for power strategies, irrespective of both NPMs, were significantly higher (*P*<.05) for most countries during PVT than non-PVT, with the highest rate observed for the Philippines (WPRO: 8.91 advertisements per hour per channel and SEARO: 8.82 advertisements per hour per channel) and the lowest for Malaysia (WPRO: 1.88 advertisements per hour per channel and SEARO: 1.84 advertisements per hour per channel). In contrast, rates of power strategies for not permitted foods in China and Nepal were significantly higher during non-PVT compared to PVT when classified using WPRO criteria, whereas power strategy rates in India remained similar during PVT and non-PVT irrespective of both NPMs.

When probing premium offers given for not permitted food advertisements, rates were significantly higher (*P*<.05) during PVT compared to non-PVT for most countries by either WPRO or SEARO criteria. Rates were the highest for Bangladesh (WPRO: 1.59 advertisements per hour per channel and SEARO: 1.59 advertisements per hour per channel) and lowest for China (WPRO: 0.06 advertisements per hour per channel and SEARO: 0.05 advertisements per hour per channel) irrespective of both NPMs. No premium offers were broadcast during PVT and non-PVT on Nepal television.

**Table 3:** Persuasive strategies in not permitted food advertisements during peak and nonpeak viewing times.

Country	Not permitted food advertisement rates^a^ (advertisements per hour per channel)														
	Power strategies								Premium offers						
	WPRO^b^				SEARO^c^				WPRO				SEARO		
	PVT^d^, mean (SD)	Non-PVT, mean (SD)	*P* value	PVT, mean (SD)		Non-PVT, mean (SD)	*P* value	PVT, mean (SD)		Non-PVT, mean (SD)	*P* value	PVT, mean (SD)		Non-PVT, mean (SD)	*P* value
Bangladesh	5.24 (4.45)	3.28 (3.57)	<.001	3.31 (3.57)		1.97 (3.12)	<.001	1.59 (2.22)		0.58 (1.52)	<.001	1.59 (2.22)		0.58 (1.52)	<.001
China	2.71 (2.92)	3.56 (6.64)	.02	3.71 (4.74)		4.51 (7.60)	.05	0.06 (0.26)		0.05 (0.25)	.13	0.05 (0.24)		0 (0)	<.001
India	5.06 (3.16)	5.21 (4.00)	.88	4.30 (2.74)		4.08 (3.20)	.11	0.49 (0.86)		0.47 (0.92)	.43	0.40 (0.77)		0.33 (0.84)	.005
Malaysia	1.88 (2.87)	0.82 (1.49)	<.001	1.84 (2.90)		0.80 (1.42)	<.001	0.77 (1.14)		0.36 (0.78)	<.001	0.76 (1.10)		0.34 (0.71)	<.001
Mongolia	7.06 (7.30)	4.64 (5.96)	<.001	6.90 (6.89)		4.68 (5.81)	<.001	0.95 (1.64)		0.38 (0.96)	<.001	0.92 (1.42)		0.54 (1.04)	<.001
Nepal	2.06 (2.53)	2.40 (2.71)	.049	1.77 (2.24)		1.94 (2.23)	.12	0 (0)		0 (0)	—^e^	0 (0)		0 (0)	—
Philippines	8.91 (7.88)	6.38 (6.83)	<.001	8.82 (7.77)		6.32 (6.71)	<.001	0.53 (0.76)		0.44 (0.78)	.009	0.53 (0.76)		0.44 (0.78)	.009
Sri Lanka	4.74 (4.02)	3.50 (3.49)	<.001	5.46 (4.07)		4.21 (3.98)	<.001	1.25 (1.67)		0.97 (1.68)	.002	1.14 (1.68)		0.75 (1.60)	<.001
Vietnam	3.98 (5.58)	1.17 (3.65)	<.001	4.17 (5.79)		1.28 (3.87)	<.001	1.10 (1.46)		0.26 (0.77)	<.001	1.07 (1.44)		0.27 (0.79)	<.001

^a^Not permitted food advertisements include solid foods and beverages. It excludes advertisements for culinary ingredients, nutritional supplements, baby food, and follow-up formula.

^b^WPRO: World Health Organization regional offices for Western Pacific.

^c^SEARO: World Health Organization regional offices for South-East Asia.

^d^PVT: peak viewing time.

^e^Not available.

### Nature of Most Frequently (%) Advertised Not Permitted Food Categories

**Figure 1:**
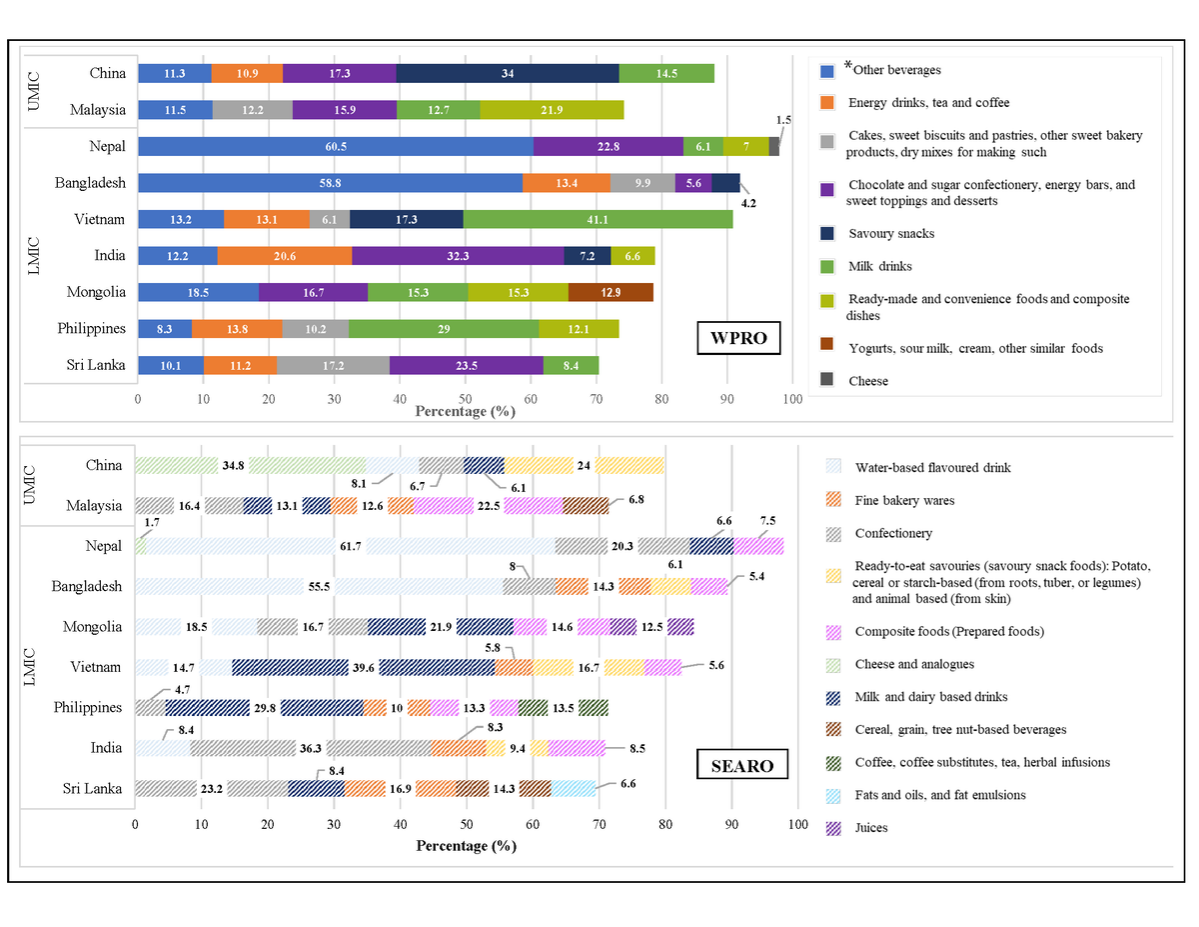
Top 5 (%) most frequently advertised not permitted foods by WPRO and SEARO criteria. LMIC: low- and middle-income countries; SEARO: World Health Organization regional offices for South-East Asia; UMIC: upper- and middle-income countries; WPRO: World Health Organization regional offices for Western Pacific. *Example of other beverages includes chocolate malt beverages, juice drinks, mineral water, and carbonated soft drinks.

We observed that the highest frequency (%) of advertisements for not permitted food categories, irrespective of both NPMs, was driven by HFSS foods and beverages (Figure 1), which varied between countries and likely reflected local popular tastes. When categorized by WPRO criteria, the highest advertisement frequency related to “other beverages” for Nepal, Bangladesh, and Mongolia; “savoury snacks” for China; “chocolate and sugar confectionery, energy bars, and sweet toppings and desserts” for India and Sri Lanka; “ready-made, convenience foods and composite dishes” for Malaysia; and “milk drinks” for Vietnam and the Philippines. When categorized by SEARO criteria, advertisement frequencies were still reflecting local taste preferences for HFSS products as per “water-based flavored drinks” for Bangladesh and Nepal, “milk and dairy based drinks” for Vietnam, the Philippines, and Mongolia; “confectionery” for India and Sri Lanka; “composite foods (prepared foods)” for Malaysia; and “cheese and analogues” for China.

**Figure 2:**
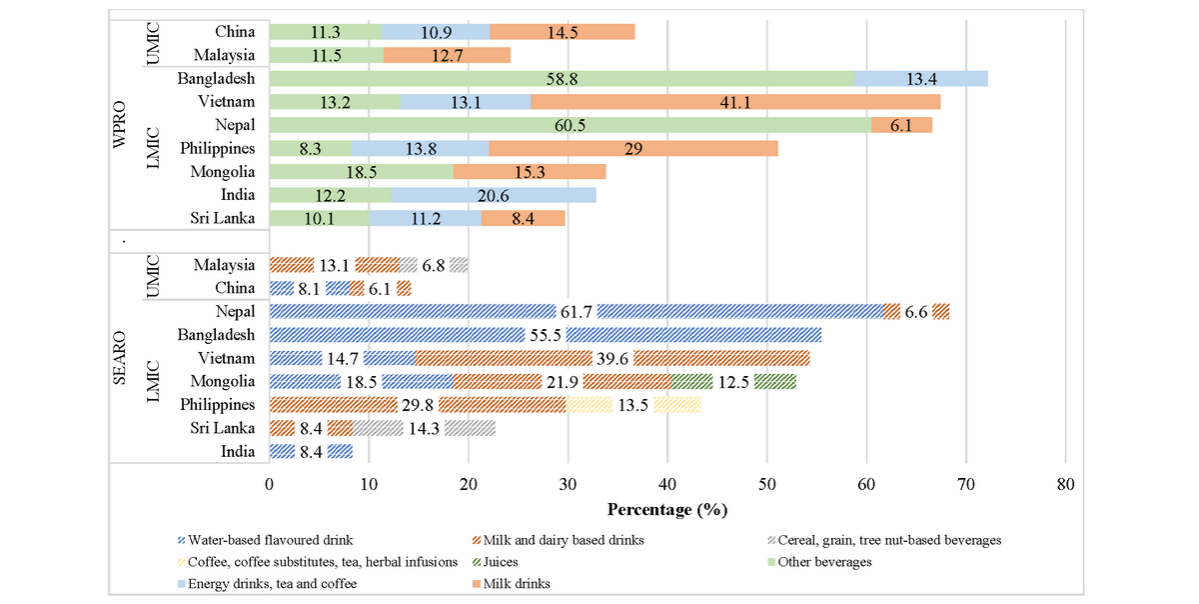
Top 5 (%) most frequently advertised not permitted products by WPRO and SEARO criteria as per combined estimates for sugar and nonsugar sweetener–based beverage products. LMIC: low- and middle-income countries; SEARO: World Health Organization regional offices for South-East Asia; UMIC: upper- and middle-income countries; WPRO: World Health Organization regional offices for Western Pacific.

We further appraised the combined share of the sugar and nonsugar sweetener–based beverage products for each country (Figure 2), which were prominent among the top 5 frequently advertised not permitted foods. As per WPRO criteria, this totaled 72.2% (n=2207) for Bangladesh, 67.4% (n=1583) for Vietnam, 66.6% (n=1607) for Nepal, 51.1% (n=3115) for the Philippines, 36.7% (n=1004) for China, 33.8% (n=2237) for Mongolia, 32.8% (n=1403) for India, 29.7% (n=1543) for Sri Lanka, and 24.2% (n=437) for Malaysia. Of note, the WPRO category for “energy drinks, tea and coffee” included beverages marketed without sweetening agents such as teas and coffees, which likely targeted adult consumers. With SEARO criteria, these data changed: 68.3% (n=1527) for Nepal>55.5% (n=1180) for Bangladesh>54.3% (n=1320) for Vietnam>52.9% (n=3506) for Mongolia>43.3% (n=2573) for the Philippines>22.7% (n=1185) for Sri Lanka>19.9% (n=348) for Malaysia>14.2% (n=540) for China>8.4% (n=277) for India.

**Figure 3:**
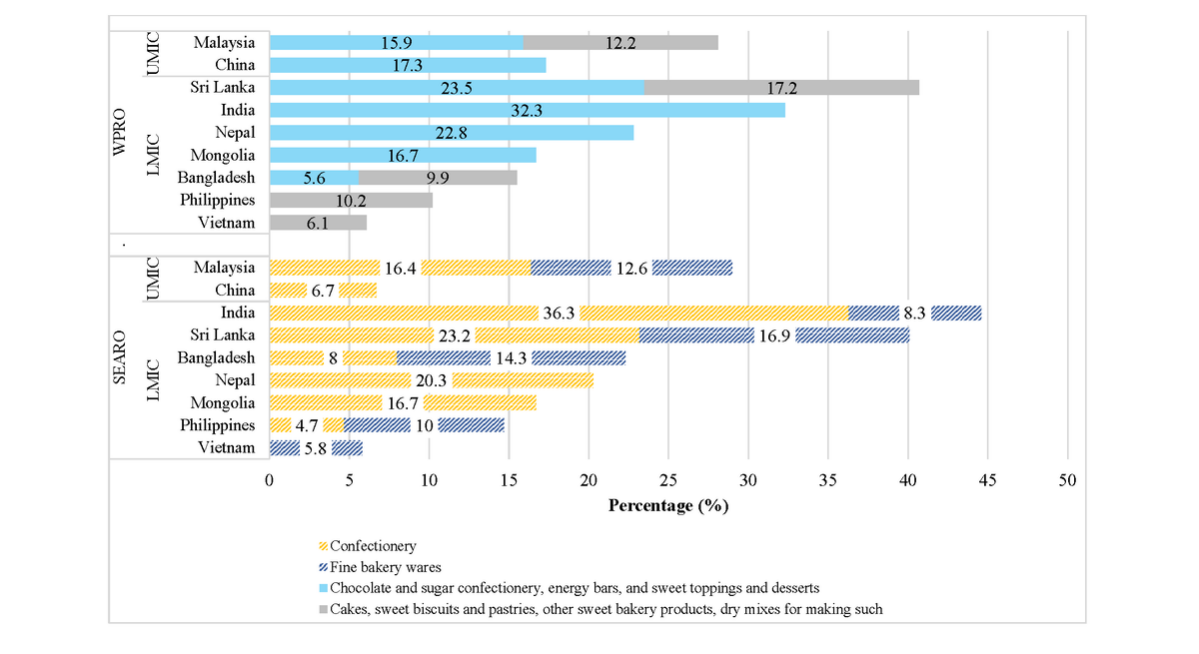
Top 5 (%) most frequently advertised not permitted products by WPRO and SEARO criteria as per combined estimates for sugar-concentrated solid food products. LMIC: low- and middle-income countries; SEARO: World Health Organization regional offices for South-East Asia; UMIC: upper- and middle-income countries; WPRO: World Health Organization regional offices for Western Pacific.

High sugar-containing foods were also frequently advertised (Figure 3), with the combined share as per WPRO criteria being the highest for Sri Lanka (n=2103, 40.7%)>India (n=1384, 32.3%)>Malaysia (n=1384, 28.1%)>Nepal (n=550, 22.8%)>China (n=472, 17.3%)>Mongolia (n=1105, 16.7%)>Bangladesh (n=474, 15.5%)>the Philippines (n=621, 10.2%)>Vietnam (n=144, 6.1%). With SEARO criteria, some differences were indicated with India (n=1466, 44.6%)>Sri Lanka (n=2093, 40.1%)>Malaysia (n=507, 29%)>Bangladesh (n=474, 22.3%)>Nepal (n=454, 20.3%)>Mongolia (n=1105, 16.7%)>the Philippines (n=869, 14.7%)>China (n=254, 6.7%)>Vietnam (n=141, 5.8%).

**Figure 4:**
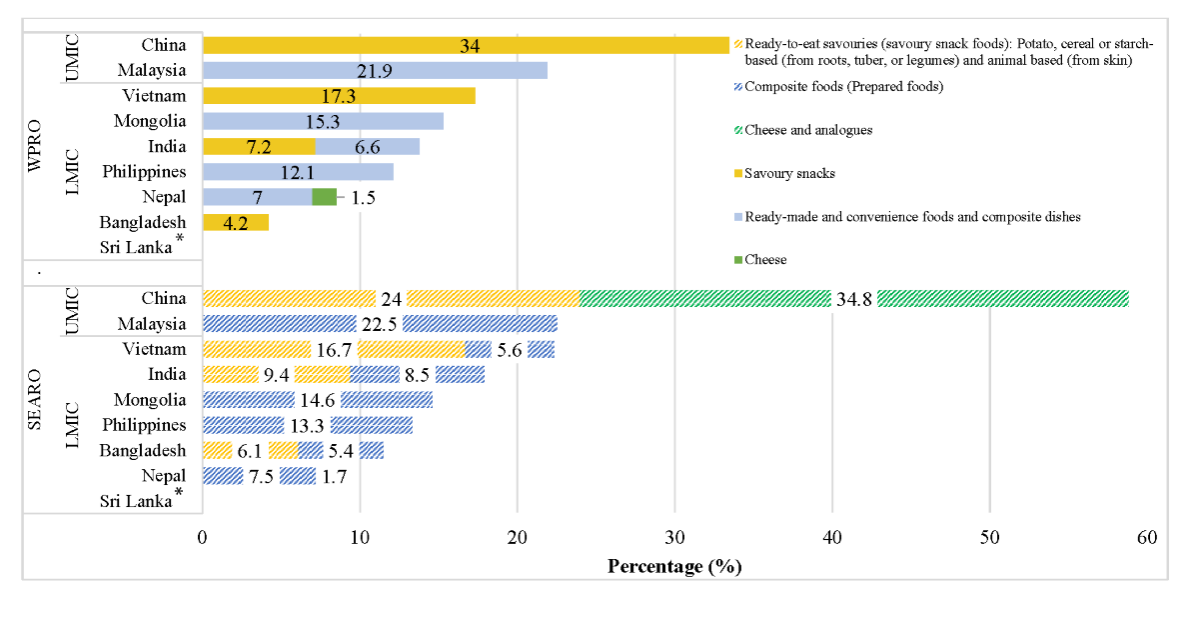
Top 5 (%) most frequently advertised not permitted products by WPRO and SEARO criteria as per combined estimates for high sodium- and fat-containing food products. LMIC: low- and middle-income countries; SEARO: World Health Organization regional offices for South-East Asia; UMIC: upper- and middle-income countries; WPRO: World Health Organization regional offices for Western Pacific. *Relevant products were not recorded.

Not permitted food advertisements characterized by high-sodium and high-fat content as per both NPM thresholds were among the top 5 frequently advertised foods in China. These were identified as “savoury snacks” (n=931, 34%) under WPRO, whereas “cheese and analogues” (n=1323, 34.8%) and “ready-to-eat savouries (savoury snack foods): potato, cereal or starch-based (from roots, tuber, or legumes) and animal based (from skin)” (n=911, 24%) were products categorized under SEARO (Figure 4). Similarly for Malaysia, Mongolia, and the Philippines, high advertising frequency trends were observed under WPRO criteria relating to “ready-made and convenience foods and composite dishes” and under SEARO criteria relating to “composite foods (prepared foods).”

## Discussion

### Principal Findings

Rapid economic growth occurring in Asia over the last decades, accompanied by nutrition transition, has unfortunately seeded childhood obesity in many countries. This phenomenon of obesity is driven through the globalization of trade and the growth of transnational and regional food businesses, which nurtured increased access to unhealthy foods and beverages [9,42-44]. Much of the food manufactured for the Asian market promotes HFSS foods and beverages, and the extent of their marketing channeled through children’s settings such as popular television was little understood in Asia [11,24,25,29,31,45]. This research provides the first baseline evidence on the extent and nature of unhealthy television food marketing to children and adolescents across 9 Asian countries (Bangladesh, China, India, Malaysia, Mongolia, Nepal, the Philippines, Sri Lanka, and Vietnam) using the uniform approach of INFORMAS [35]. Participating countries representing LMICs at various stages of epidemiological, demographic, and nutrition transitions presented an opportunity to compare and contrast the extent and type of marketing. Policy commitments to regulate unhealthy food marketing on television and other media are not mandatory in most Asian countries [27], and industry commitments to self-regulate appear to be the norm for some of the countries participating in this study.

Using INFORMAS methodology to harmonize measurement approaches, we showed that unhealthy foods, defined as “not permitted” by WHO criteria for the NPMs established for the WPRO and SEARO regions in Asia [40,41], overwhelmingly dominated food advertising through children’s popular television channels in the 9 Asian countries studied. The advertisement rate was highly varied between countries, ranging from almost 10 “not permitted” food advertisements per hour in the Philippines and Sri Lanka to 2 per hour in Malaysia. The rates observed in this study were lower than an earlier cross-country study in the Asia-Pacific region in 2014, which used a different food classification system to rate “noncore” (unhealthy) food advertising, reported rates ranging from 2.3 advertisements per hour per channel for South Korea to 16.7 advertisements per hour per channel for Indonesia [31]. This finding aligns with studies from Slovenia, Chile, and Thailand, which, in benchmarking food advertisements to standards of perceived thresholds for the “danger” HFSS nutrients, detected a high concentration of unhealthy food advertisements on children’s popular television channels [46-48].

With the exception of India, China, and Nepal, most countries observed higher rates of not permitted food advertisements during the broadcast periods most popular with children compared to non-PVTs. This aligns with earlier studies [31,39,49], which also found higher rates of unhealthy food advertising during children’s PVTs. Uniquely, China had significantly lower rates of not permitted food advertisements during children’s PVTs compared to non-PVTs based on the SEARO classification. This may be attributed to local advertising regulations in China, which require several advertising slots to be reserved for public service advertisements (including healthy food such as fresh fruit) during these broadcast periods [50].

The use of persuasive marketing techniques embedded in not permitted food advertisements was also significantly higher during children’s PVTs in most of the participating countries, which also concurs with previous multicountry studies [31,39] showing higher rates of promotional characters and premium offers during children’s PVTs compared to non-PVTs. Taken together, the higher rate of unhealthy food marketing during popular broadcast times for children and the higher use of persuasive marketing techniques that appeal to children during these broadcast times clearly suggest advertisers are targeting children with unhealthy food advertising.

As this study used both the WPRO and SEARO NPM to classify advertised foods, it became possible to explore differences in the performance of these systems in categorizing food marketing across Asian countries. SEARO is the NPM applicable to India, Nepal, Sri Lanka, and Bangladesh, whereas WPRO is applicable to China, Malaysia, Mongolia, the Philippines, and Vietnam. Prior to this study, most studies investigating content analyses [12,39,51] related to the WHO model of the Europe Regional Office [52]. Applicable to the Asian scenario, this study therefore is the first to relate content analyses of food advertising to WPRO [40] and SEARO NPMs [41].

Application of the SEARO model compared to the WPRO model led to a lower ratio of “permitted” to “not permitted” advertised foods for most countries, except China and Sri Lanka, where ratios were comparable between the 2 NPMs. Differences occurred when healthy food and beverage items such as plain mineral water were included under WPRO but not included in the SEARO model. Another reason for higher ratios associated with WPRO was the exclusion of culinary ingredients, which otherwise would be identified as permitted food advertisements.

Upon further comparing WPRO and SEARO criteria, the fewer beverage categories available under WPRO lead to more products to be accommodated within a category. For example, both carbonated and chocolate malt drinks are grouped under “other beverages.” Whereas under SEARO, these beverages are grouped separately as carbonated drinks under “water-based flavoured drinks” and chocolate malt beverage products under “cereal, grain, tree nut-based beverages.” In scrutinizing the combined share of beverages among the top 5 frequently advertised not permitted foods, we noted higher frequencies for India and China using WPRO criteria for 2 categories, namely “energy drinks, tea and coffee” and “other beverages.” However, the single beverage category of the SEARO criteria, vis à vis “water-based flavoured drink,” generated lower frequencies when combining the top 5 beverages. Food advertisements categorized under “energy drinks, tea and coffee” as per WPRO criteria would be considered as not permitted with or without sugar content [40]. Whereas a total sugar threshold of up to 2 g would be permitted for categories such as “coffee, coffee substitutes, tea, herbal infusions” and “water-based flavoured drink” (which included energy drinks) or up to 6 g for “cereal, grain, tree nut-based beverages” under the SEARO criteria [41]*.*

Even when considering sugar-concentrated solid food products, differences between NPMs could be explained by categories such as “chocolate and sugar confectionery, energy bars, and sweet toppings and desserts” as well as “cakes, sweet biscuits and pastries, other sweet bakery products, dry mixes for making such” were not allowed to be marketed under WPRO regardless of sugar level [40]. Whereas SEARO allowed a total sugar threshold of up to 6 g [41]. Contrarily, the “cheese” category under SEARO, despite identifying foods with high-sodium and high-fat thresholds as not permitted, includes an additional criterion of “zero” tolerance for added sugar [40,41].

Advertising of fast foods is a concern for Malaysia, Mongolia, and the Philippines with high advertising frequency trends for “ready-made and convenience foods and composite dishes” under WPRO and composite foods (prepared foods) under SEARO criteria. The implication for Malaysia, in particular, is a failure of a self-regulatory policy known as the *Guideline on the Advertising and Nutrition Information Labelling of Fast Foods* [53,54]. This guideline was introduced in 2008 to restrict advertising of fast food during children’s programs if ≥4% of children aged 4-9 years comprise the television viewing audience. However, the guideline is not legislated, and government-led monitoring for the guideline is lacking [54].

A trait of television advertising in Asia is featuring culinary ingredients even during PVTs [24,26,45]. It is noted that PVT equates to prime time for Asian families, running between 6 and 11 PM, which also attracts the viewership of the whole family. It is not surprising that this period also carries advertisements for culinary ingredients targeting women, as women traditionally purchase foods for their household, cook foods, and decide what the family consumes [55]. Additionally, labels of culinary items featured in advertisements will not provide serving size, as they are ingredients combined with other ingredients in cooking meals. Unique culinary practices in the Asian region follow recipes prepared in the home requiring many staples (eg, uncooked rice, cooking oil, spices, seasoning powder, uncooked meat, chicken or vegetable stock, recipe premix, plain sugar, stevia, plain flour, breading mix, lentils, and coconut milk). For Malaysia and Vietnam, items such as uncooked rice and soybean cooking oil would otherwise be identified as permitted foods as per WPRO criteria (Table S2 in Multimedia Appendix 1). We, therefore, excluded culinary ingredients from our analyses.

### Strength and Limitations

A major strength of this study was the harmonized measurement approaches adopted by all 9 Asian countries, enabled comparisons between countries using 2 WHO NPMs for determining the extent, intensity, and nature of unhealthy food marketing on television. The comparative assessment of WPRO and SEARO NPMs across the 9 Asian countries facilitated the identification of inconsistencies in their application to categorize food advertisements due to regional and cuisine differences, thereby indicating the need for improvement across these 2 models. Overall, the findings from this study contribute evidence of the need to strengthen local regulatory frameworks in each country to stimulate advocacy to stakeholders. Of priority, there is an urgent call for mandatory policy action in Asian countries to restrict children’s exposure to unhealthy food marketing on television to protect them from the harmful impact of food marketing [6]. Ultimately, a comprehensive regulatory framework with consistent monitoring over time should enable the reduction of exposure and power of unhealthy food marketing directed to children. A further benefit in conducting this study was that the collaboration achieved between country teams and INFORMAS experts enabled the exchange of technical knowledge and building the capacity of researchers across the 9 Asian countries.

This study’s protocol required recording advertisements shown on the top 3 children’s popular television channels for each country rather than measuring actual children’s media exposure. The latter approach to measure actual targeted media exposure of children to television is experimental, with behavioral research methodology being varied and challenging to execute in multicountry scenarios where a harmonized protocol in low-resource settings is critically required [56]. Second, as this study was initiated during the COVID-19 lockdown occurring in all countries, we adopted convenience sampling to accommodate permitted travel days to the recording site. However, COVID-19 may have changed the nature of advertising, as this period may not have reflected the usual advertising pattern. Coding challenges for food products were encountered where countries lacked mandatory labeling regulation, such as Vietnam. In such cases, researchers would refer to their country’s or the neighboring country’s food composition database.

### Future Directions

Future research on television food marketing in individual countries should consider recording more television channels, such as in India and China. Ideally, randomized sampling for recording days should be adopted, but randomization should be appropriate to the cultural diversity and practices in Asia. Given this successful collaboration between the Asian countries on television food marketing, there is now a need for explorative research on the digital food marketing landscape in Asia, as the food industry has expanded its marketing strategy to digital media platforms.

### Conclusions

This project’s collaboration and use of harmonized methodology generated country-level evidence for 9 Asian countries on the exposure and power of unhealthy food marketing on television to children. Cross-country comparisons, irrespective of country income level, indicated that unhealthy food advertising dominated children’s popular television channels with frequent advertising of sugar-sweetened beverages, sugar-containing solid food and snacks, and fast foods high in salt and fat, especially during PVTs. Clearly, unhealthy food marketing through popular children’s television channels is occurring widely in Asia and is a clear breach of child rights. Evidence outcomes could be used to advocate for stronger policy regulations and implementation to control unhealthy food marketing, which will strengthen strategies promoting a healthier food environment for Asia’s children.

## Appendix

Multimedia Appendix 1 Supplementary tables.

## Abbreviations

HFSS: high in fat, sugar, and salt
INFORMAS: International Network for Food and Obesity/Non-Communicable Disease Research, Monitoring and Action Support
IRR: intercoder reliability
LMICs: low- and middle-income countries
NCD: noncommunicable disease
NPM: nutrient profile model
PMT: project management team
PVT: peak viewing time
SEARO: World Health Organization regional offices for South-East Asia
WHO: World Health Organization
WPRO: World Health Organization regional offices for Western Pacific

## Data Availability

The data sets generated and analyzed during this study are available from the corresponding author on reasonable request.
