# Supplementary material for: Extent and Nature of Television Food and Nonalcoholic Beverage Marketing in 9 Asian Countries: Cross-Sectional Study Using a Harmonized Approach
Source: JMIR Pediatr Parent. 2024 Dec 4;7:e63410. doi: 10.2196/63410 (PMC11656118; doi:10.2196/63410)
Supplement: Multimedia Appendix 1 [file pediatrics_v7i1e63410_app1.docx]

**Table S1.** Example of culinary ingredients.

| **Country** | **Example of culinary ingredients** | | | |
| --- | --- | --- | --- | --- |
|  | **WPRO** | | **SEARO** | |
|  | **Permitted** | **Not Permitted** | **Permitted** | **Not Permitted** |
| Bangladesh | Mustard oil, Soybean oil, Sunflower oil, Plain white rice, Plain flour, Spices | Seasoning powder | Mustard oil, Soybean oil, Sunflower oil, Plain white rice, Plain flour, Spices | Seasoning powder |
| China | Sunflower oil, Peanut oil, Canola oil, Soybean oil, Walnut oil, Plain white rice, Fresh mutton meat, Fresh beef, Fresh Potatoes, Paprika powder, Black & White pepper powder | Chicken flavouring powder/ stock, | Sunflower oil, Peanut oil, Canola oil, Soybean oil, Walnut oil, Plain white rice, Fresh mutton meat, Fresh beef, Fresh Potatoes, Paprika powder, Black & White pepper powder | Chicken flavouring powder/ stock, |
| India | Olive oil, Mustard oil, Sunflower oil, Plain flour, Plain white rice, Lentils, Spices, | Stevia, Soybean oil^a^, Ghee, Corn and rice bran blended oil^a^, Salt, Ginger garlic paste, Seasoning powder, | Olive oil, Mustard oil, Soybean oil, Sunflower oil, Corn and rice bran blended oil, Plain flour, Plain white rice, Lentils, Spices, | Stevia, Ghee, Salt, Ginger garlic paste, Seasoning powder, |
| Malaysia | Plain white rice, Plain flour | Seasoning powder/ cubes, Premixed sambal sauce | Plain white rice, Plain flour, Seasoning cubes | Seasoning powder, Premixed sambal sauce |
| Mongolia | Sunflower oil, Plain flour, Fresh chicken meat, Fresh lamb, Fresh pork, Fresh vegetable | - | Sunflower oil, Plain flour, Fresh chicken meat, Fresh lamb, Fresh vegetable | Fresh pork |
| Nepal | Sunflower oil, Plain flour, Plain white rice, Lentils | - | Sunflower oil, Plain flour, Plain white rice, Lentils | - |
| Philippines | - | Breading mixes, Seasoning powder/ paste, Chicken/beef flavoured evaporated creamer for cooking, Recipe premix, Bouillon cubes, | - | Breading mixes, Seasoning powder/ paste, Chicken/beef flavoured evaporated creamer for cooking, Recipe premix |
| Sri Lanka | Plain white rice, Fresh vegetable, Lentils, Spices | White sugar, Salt, Seasoning powder/ cubes | Plain white rice, Fresh vegetable, Lentils, Spices, Seasoning cubes | White sugar, Salt, Seasoning powder/ cubes |
| Vietnam | Soybean oil, Plain white rice | Rice bran oil^a^, Seasoning powder, Breading mixes, | Soybean oil, Plain white rice, Rice bran oil | - |

^a^Saturated fat content (SFA) declared on the packaging exceeded the WPRO’s SFA threshold of 20g.

**Table S2.** Rates of Culinary Ingredients Ads Rates as per WPRO and SEARO criteria.

| **Country** | **Culinary Ingredients Ads Advertisement Rates**  **Ads/h/Channel (mean** ±**SD)** | | | | |
| --- | --- | --- | --- | --- | --- |
|  | All Culinary Ingredients Ads^a^ | Culinary Ingredients Ads | | | |
|  |  | WPRO | | SEARO | |
|  |  | Permitted | Not Permitted | Permitted | Not Permitted |
| Bangladesh | 1.21 ± 2.22 | 0.92 ± 1.88 | 0.29 ± 0.98 | 0.92 ± 1.88 | 0.29 ± 0.98 |
| China | 0.74 ± 1.39 | 0.62 ± 1.21 | 0.11 ± 0.46 | 0.62 ± 1.21 | 0.11 ± 0.46 |
| India | 0.62 ± 1.15 | 0.22 ± 0.70 | 0.37 ± 0.75 | 0.30 ± 0.83 | 0.29 ± 0.69 |
| Malaysia | 0.12 ± 0.51 | 0.07 ± 0.46 | 0.05 ± 0.24 | 0.08 ± 0.47 | 0.04 ± 0.20 |
| Mongolia | 1.06 ± 1.76 | 1.06 ± 1.76 | 0 | 0.53 ± 1.28 | 0.53 ± 1.42 |
| Nepal | 0.57 ± 1.32 | 0.57 ± 1.32 | 0 | 0.57 ± 1.32 | 0 |
| Philippines | 1.10 ± 1.68 | 0 | 1.03 ± 1.56 | 0 | 1.03 ± 1.56 |
| Sri Lanka | 1.59 ± 2.06 | 0.41 ± 1.05 | 1.17 ± 1.64 | 0.82 ± 1.40 | 0.76 ± 1.34 |
| Vietnam | 0.22 ± 0.76 | 0.08 ± 0.35 | 0.14 ± 0.67 | 0.09 ± 0.37 | 0 |

^a^Includes all culinary ingredients rated as “permitted” and not permitted” as per the WPRO and SEARO criteria as well as items lacking sufficient nutrition information for rating against the NPM profile.
